# Accumulation of selected metals and concentration of macroelements in liver and kidney tissues of sympatric golden jackal (*Canis aureus*) and red fox (*Vulpes vulpes*) in Somogy County, Hungary

**DOI:** 10.1007/s11356-021-15156-y

**Published:** 2021-07-08

**Authors:** Attila Farkas, András Bidló, Bernadett Bolodár-Varga, Ferenc Jánoska

**Affiliations:** 1 Faculty of Technical and Human Sciences, Sapientia Hungarian University of Transilvania, Corunca, 1C, 540485 Târgu-Mureș, Romania; 2 Faculty of Forestry, Institute of Environmental and Earth Sciences, University of Sopron, Bajcsy-Zs. str. 4, Sopron, H-9400 Hungary; 3 Faculty of Forestry, Institute of Wildlife Management and Vertebrate Zoology, University of Sopron, Bajcsy-Zs. str. 4, Sopron, H-9400 Hungary

**Keywords:** Golden jackal, Red fox, Liver, Kidney, Biomonitor, Metals, Macroelements, Hungary

## Abstract

This study examined the concentrations of Al, Ca, Cd, Cr, Cu, Fe, K, Mg, Mn, Na, Ni, Pb, and Zn in the liver and kidney samples of golden jackals (n = 163) and red foxes (n = 64). Additionally, we studied how sex affected element concentration in both species, and in the case of golden jackals, how concentration levels were affected by age.

Liver was the most important storage organ for Cu, Fe, Mn, and Zn, while kidney for Cd and Na in jackals and foxes.

We found no sex-related differences in concentration of any of the selected elements in fox samples. In jackals, the mean concentrations of Al, Mn, and Zn were significantly higher in kidneys of males, while Zn and Cu had higher concentrations in female livers. Our results suggest bioaccumulation processes do not occur in mesocarnivores.

Lead and Cd concentrations fell within ranges accepted as normal for canine species and were below toxic levels in the organs of the examined species. Concentrations of Cr, Cu, Fe, Mn, and Zn were far below the toxic levels in both organs of both species, with a slight sign of deficiency of Fe, Mn, and Zn in jackal and fox kidneys. In comparison with toxicity levels and results from other European study sites, the current study demonstrated that species living in the examined area of Somogy County, Hungary, are generally exposed to low levels of environmental contamination.

**Supplementary Information:**

The online version contains supplementary material available at 10.1007/s11356-021-15156-y.

## Introduction

Many chemical element concentrations present in nature stem from human activity; moreover, some concentrations raise concerns about environmental contamination (Tchounwou et al. 2012). Nevertheless, many contaminants found in the environment occur naturally. Contamination can be detected in soil, rainwater, and living or deceased organisms (Nordberg et al. 2015; Kalisińska 2019a; Keresztesi et al. 2019). Some habitats have different levels of contamination (e.g., Zietara et al. 2019), but the concentrations of chemical elements cannot be clearly associated with small, local, or industrial emitters (Sedláková et al. 2019). At the European scale, Reimann et al. (2018) found that the variation in the natural background concentration of 53 elements (including 14 potentially toxic ones) in the agricultural soil samples is much larger than any anthropogenic impact. Nonetheless, evidence of significant remote cross-border pollution is steadily increasing as well (Keresztesi et al. 2020). Due to the variety of possible sources of environmental contaminants, it would be more effective to focus biomonitoring studies on targeted living organisms across selected habitats rather than merely focus on uncertain or suspected sources of environmental contamination.

We selected 12 chemical elements and studied their concentrations in a Hungarian study site. No major sources of pollution, such as metal industry factories or coal-fired plants, operate near or within the study area. The list of selected elements included seven that Ali and Khan (2018) categorize as heavy metals: cadmium—Cd, chromium—Cr, copper—Cu, iron—Fe, manganese—Mn, lead—Pb, and zinc—Zn; 4 minerals (calcium—Ca, potassium—K, magnesium—Mg, and sodium—) or macroelements (Pohl et al. 2019); and aluminum (Al), the third most abundant element in nature (Skibniewska and Skibniewski 2019). Element concentration studies were performed on liver and kidney samples of golden jackals (*Canis aureus*) and red foxes (*Vulpes vulpes*). These two species fulfill all criteria to serve as biomonitors including the following: their large geographical distribution, limited feeding range, position on the top of the local food chain, relatively long life span, and easy sampling via regular hunting activities (Dip et al. 2001; Binkowski et al. 2016; Kalisińska 2019b).

Though the usage of mesopredators in biomonitoring and ecotoxicological studies is well-founded, only one study in this topic has been completed thus far in Hungary. This study established the mean concentration values of 6 metal residuals (Cu, Ni, Zn, Co, Cd, and Pb) in red fox livers and kidneys and defined the initial norm of their variation for an agricultural landscape (Heltai and Markov 2012). We considered that simultaneous residual analyses of two organ samples collected from two sympatric mesocarnivore species could lead to a solid contribution to the field of European ecotoxicological studies. Hence, our aims were as follows: (1) to set initial concentration values of the selected elements in Somogy County, Hungary; (2) to investigate the species-related and organ-dependent concentrations; (3) to test the effects of sex and age groups; and (4) to contextualize our results with other European studies performed on golden jackals and red foxes.

## Materials and methods

### Study area

**Fig. 1:**
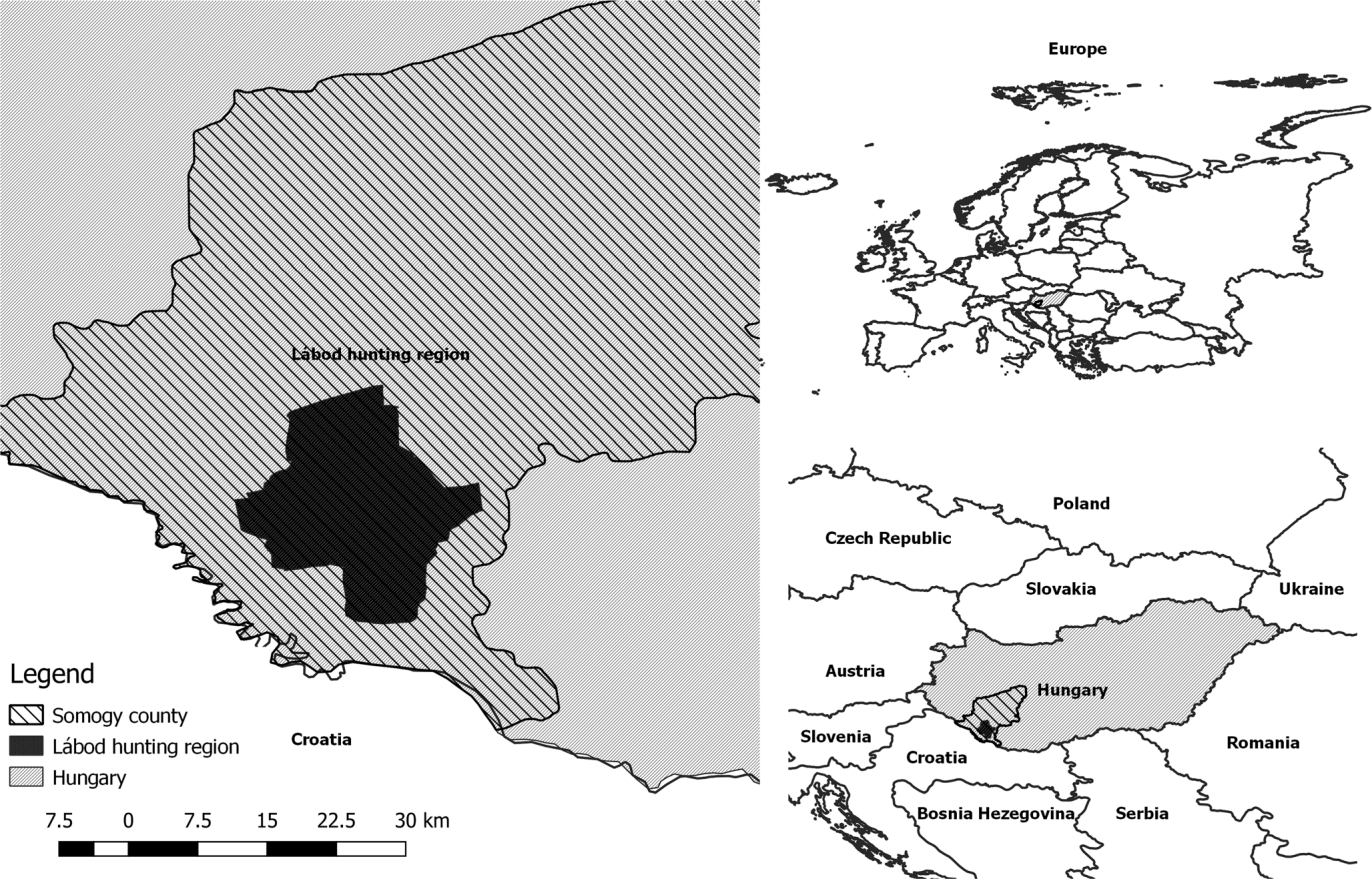
Map of study area, Lábod hunting region (Hungary)

Somogy County is part of the Southern Transdanubia statistical region (NUTS 2) of Hungary. The study area is the Lábod hunting region (centre: 46° 9′59.76″ N, 17°27′16.30″ E; 48,200 ha; Fig. 1.) located within the county and managed by one of the Hungary’s 22 state-owned forestry companies, SEFAG J.S.C.

The location is lowland area with sand dunes (130–160 m a.s.l); the climate is sub-Mediterranean; the mean multiannual temperature is 10.2°C; the annual number of days with snow cover is 30–34, with an average snow depth of 6–10 cm. The average annual precipitation is between 700 and 800 mm. Land cover structure, calculated based on the Corine Land Cover 2018 Habitat Map compiled by the European Environment Agency (EEA), show that forests and seminatural areas (CLC code = 311, 312, 313, 321, 324) cover 56.11%, agricultural areas (CLC code = 211, 222, 231, 242, 243) occupied 39.83%, wetlands and water courses (CLC code = 411, 512) 1.58%, while artificial surfaces (CLC code = 112, 121, 142) cover 2.48% of the area. Forest vegetation consists of 25.5% alder (*Alnus*) species, 20.7% English oak (*Quercus robur*), 19% black locust (*Robinia pseudoacacia*), and 10.9% Scots pine (*Pinus sylvestris*). The remainder is constituted mainly of willow (*Salix*) and linden (*Tilia*) species (Nagy et al. 2014; Lanszki et al. 2015).

Wildlife management is based primarily on the five big game species present in Hungary, namely red deer (*Cervus elaphus*), fallow deer (*Dama dama*), roe deer (*Capreolus capreolus*), mouflon (*Ovis aries*), and wild boar (*Sus scrofa*). Golden jackals (*Canis aureus*) and red foxes (*Vulpes vulpes*) fulfill the roles of apex predator species in this area. The Hungarian Game Management Database (Csányi et al. 2018) documents that 2965 red foxes and 1668 golden jackals were harvested during the 2017/2018 hunting season. Such hunting bags are equivalent to 0.48 red foxes/km^2^ and 0.27 golden jackals/km^2^. The past two decades have seen significant exponential trends of population assessment data for jackal, while linear growth trends for foxes remain typical (Banea et al. 2018).

According to the Somogy Chamber of Commerce and Industry, Somogy County has a population density of 50 inhabitants per km^2^, making it the most sparsely populated and economically underdeveloped county in Hungary, producing only 2% of Hungary’s GDP. With relatively high forest cover and an economy based on forestry, wildlife management, agriculture, and tourism, Somogy is considered the “green heart” of Hungary. However, the study area cannot be considered a pristine natural environment and is not free of agriculture and traffic, which could be the main sources of chemical and metal pollution (Kalisińska 2019b).

### Sample collection

From April 2017 to January 2018, we collected liver and kidney samples from 64 red foxes and 163 golden jackals in the Lábod hunting region (Table 1). All specimens were hunted via legal methods (driven hunts, stalking, and blind and stand hunting), in some cases with use of big game viscera as bait. Information about sex, weight, age group, and date of harvest were recorded for each specimen. Sex was determined based on visual examinations of genital organs. Hunted specimens with juvenile features and deciduous dentition in the period of August–October were categorized as juveniles, under 1 year of age; those with adult body sizes and permanent teeth were classified as adults, having at least- or above 1 year of age.

**Table 1:** Number of collected organ samples grouped by animal’s age group and sex

Species	Age group	Sex	Organ samples (N)			
			Liver	Kidney	Liver+kidney^*^	Total
Red fox	Adult	Male	23	5	8	36
		Female	14	1	13	28
		Total	37	6	21	64
Golden jackal	Adult	Male	62	5	19	86
		Female	33	4	18	55
		Total	95	9	37	141
	Juvenile	Male	8	0	0	8
		Female	14	0	0	14
		Total	22	0	0	22
	All groups (jackal)		117	9	37	163
All groups (fox+jackal)			154	15	58	227

*Animals with both tissues sampled

All foxes and jackals were in normal physical condition, and during the autopsy, we excluded from the sample collection the specimens whose kidney or liver was affected by ammunition. The liver and kidney samples were collected, packaged, and labeled immediately after the hunts and then kept frozen at −20°C until further laboratory analysis.

### Element analysis

Laboratory analyses were performed at the Institute of Environmental and Earth Sciences within the Faculty of Forestry at the University of Sopron, Hungary. The kidney and liver samples were collected randomly and dried out to constant weight in an oven at 70°C. The moisture content of sampled organs was not measured. After drying, the samples were ground using porcelain mortar. About 0.5 g of each sample was measured in a Teflon bomb and then treated by adding 5 ml of 69% HNO_3_ and 2 ml of 30% H_2_O_2_. Teflon bombs were put in an oven at 110°C for 3 h during which the samples decomposed. The samples were washed in 50 ml volumetric flasks, from which we determined the targeted element concentrations directly using iCAP 6300 Duo View ICP-OES spectrometer (Thermo Fisher Scientific Inc, Waltham, MA, USA). All chemicals used were of analytical grade. During sample preparation and measurement, ultrapure water was used according to ASTM D1193–Standard Specification for Reagent Water (type 1). Certified reference materials were not used. The detection limits of studied elements for both liver and kidney samples were as follows: Al—0.391, Ca—1.902, Cd—0.058, Cr—0.147, Cu—1.145, Fe—0.490, K—5.298, Mg—0.589, Mn—0.082, Na—3.862, Pb—0.161, and Zn—5.244. The concentrations of the analyzed elements were expressed in milligrams per kilogram dry weight (mg kg^−1^ dw). Recovery limits were as follows: Al—100%, Ca—98%, Cd—104%, Cr—96%, Cu—100%, Fe—99%, K—102%, Mg—102, Mn—98%, Na—98%, Pb—101%, and Zn—104%.

### Statistical analysis

Basic statistical parameters (the mean and the standard deviation of mean values) of untransformed concentration data were calculated. Concentrations below detection limits and extreme values, those exceeding ±3 standard deviation limits (Table S1), were excluded. Detailed statistical analyses were only executed on valid data (Table 2 and S2). Distribution of concentration values of selected elements in liver and kidney tissues of golden jackals and red foxes was checked for normality using Kolmogorov-Smirnov D-statistics (Table S3 and S4). The homogeneity of variances among species (jackal and fox) (Table S5) and tissues (kidney and liver) (Table S6 and S7) was tested using Levene’s test. Between-species comparisons of concentration values for metals in kidney and liver samples were executed using T-test for independent samples and Mann-Whitney U Test (if homogeneity of variances differed significantly, Table S5). Comparisons between levels of selected element concentrations in fox and jackal tissue samples in kidneys and livers were performed based on the full set of data as well as on sample pairs (organs of the same specimens). With full datasets, comparisons were made using T-test for independent samples assuming equal or separate variance estimates according to results of previously performed Levene’s tests. Comparisons by sample pairs were performed using T-test for dependent samples in cases of homogenous variances and Wilcoxon Matched Pairs Test if variances were not homogenous (Table S6 for jackals and Table S7 for foxes). The age group effect could not be tested in jackal kidney samples or in any red fox organ samples. The effects of sex on concentrations of selected elements in kidney tissues of golden jackals (Table S8), such as in kidney and liver tissues of red foxes (Table S9), were tested using T-test for independent samples. To examine whether sex and age group affect concentration levels of the studied elements in liver tissues of jackals, we completed a parametric factorial ANOVA analysis (Table S10). All statistical analyses were carried out using STATISTICA version 13.4.0.14 (TIBCO Software Inc., USA).

**Table 2:** Concentrations of selected elements (mg/kg dw) in kidney and liver samples of red foxes (Vulpes vulpes) and golden jackals (Canis aureus)

Element	Fox						Jackal					
	Kidney			Liver			Kidney			Liver		
	N	Mean	Std. Dev.	N	Mean	Std. Dev.	N	Mean	Std. Dev.	N	Mean	Std. Dev.
Al	24	15.72	6.99	53	20.54	11.06	41	13.82	3.81	137	19.27	8.62
Ca	27	298.55	181.80	51	247.39	178.89	44	285.36	142.41	110	202.00	156.76
Cd	25	0.93	0.73	53	0.33	0.26	45	0.44	0.36	125	0.19	0.17
Cr	24	0.39	0.20	54	0.51	0.34	46	0.32	0.18	143	0.45	0.21
Cu	27	11.87	3.94	56	40.86	28.56	46	14.62	5.55	153	58.74	39.17
Fe	26	194.83	83.37	58	585.50	278.70	46	257.58	109.70	154	718.60	337.64
K	27	8499.77	2362.53	58	7898.50	1367.33	46	7941.74	1735.95	154	8123.84	1679.09
Mg	26	540.44	108.93	58	503.63	149.86	46	550.14	129.07	153	488.79	172.98
Mn	27	4.58	1.27	58	10.42	3.65	45	5.20	1.49	151	11.35	3.23
Na	27	5525.14	1736.51	58	3872.34	1247.42	46	5425.29	1620.68	152	3392.14	1105.57
Pb	26	3.74	3.27	46	7.41	8.03	40	3.44	2.84	122	5.53	5.47
Zn	26	65.96	11.74	57	112.23	31.39	46	74.08	16.23	154	110.65	28.61

## Results and discussion

### Detection of the selected elements

All 12 selected elements were detected in various concentrations both in jackals and foxes, independent of the organ (kidney or liver). In kidney samples, concentrations below detection levels (BDL) occurred in cases of four selected elements: Al (8%), Ca (1%), Cd (3%), and Cr (3%). In liver samples, Mg concentration remained undetected in less than 1% of the samples, besides those found undetected also in kidney samples: Al (3%), Ca (22%), Cd (16%), and Cr (<1%) (Table S1). In kidney samples were found relatively few (1–8%) extreme concentration values, but in livers, these values reached 7% in Cr (max. 10.42 mg/kg dw), 7% in Al (max. 1068.68 mg/kg dw), and 21% in the case of Pb (max. 26,943.26 mg/kg dw). Our investigations did not make possible the identification of possible sources of contamination, but the large percentage of extreme values of ubiquitous metals Al, Cr, and Pb, well-known contaminants, raise the need for further detailed studies targeting these elements.

### Species-related element concentrations

The trend of using the most common species of hunting interest (e.g., red fox and golden jackal) as biomonitors within a certain area in quantitative biomonitoring studies has increased significantly in the past decade (Heltai and Markov 2012; Ćirović et al. 2015; Pérez-López et al. 2015; Binkowski et al. 2016; Markov et al. 2016; Farkas et al. 2017; Georgiev et al. 2018; Zietara et al. 2019). Some of these studies focus on both sympatric red fox and the golden jackal (Farkas et al. 2017; Georgiev et al. 2018). Unfortunately, the small sample size in Georgiev et al. (2018) does not allow for relevant comparisons between concentrations of elements among jackals and foxes (N = 17 in jackals and N = 9 in foxes). During a previous study performed on samples collected in the southern part of Romania (Farkas et al. 2017), significant, species-related differences were found in the accumulations of Ca, Mg, and Mn, with higher concentrations in fox liver samples.

In kidney samples, we found significant species-related differences in concentrations of Cd, Cu, Fe, and Zn (Table S5). Higher mean concentration values were found in jackals, except for Cd whose concentrations were higher in fox kidneys. As far as we know, this is the first study to be performed on an adequate number of kidney samples, which allowed for species-related comparisons in concentration of some selected elements between jackals and foxes. In liver samples, we found higher Cd and Na concentrations in foxes, while Cu and Fe concentrations were higher in jackals. The lack of species-related differences in liver Al, Cr, and Pb found here were also reported previously in Romania (Farkas et al. 2017).

### Concentration levels in organs

The concentration levels in kidney and liver samples were tested using two approaches: the first was based on full set of samples, and the second was performed only on specimens with both organs sampled.

The concentration values of five elements (Al, Ca, Cr, Mg, and Pb) did not show distinct concentrations among kidney and liver samples of jackals, but only in the results of paired tests (Table S6). Accounting all data, we observed that the selected elements (except K) were present in differing concentrations among jackal liver and kidney samples. Hence, our results indicated that the concentration of certain elements in jackal organs (liver and kidney) could be influenced by the testing method or sample size used. Irrespective of comparison methods, we observed that Cu, Fe, Mn, and Zn were present in higher mean concentrations in jackal liver samples, while Cd and Na concentrations were higher in jackal kidneys. Depending on the testing method used, differences in accumulations of Al, Ca, Cr, Mg, and Pb in jackal kidneys and livers were inconsistent. Similar studies targeting heavy metal accumulation in both golden jackal liver and kidney samples were performed only in two Bulgarian study sites: (1) in a relatively intensive agricultural region (Markov et al. 2016) and (2) at the “Sarnena Sredna gora” mountain (Georgiev et al. 2018). Descriptive comparisons instead of statistical analyses were performed at the second location. In jackals, higher concentrations of Cu and Zn in liver samples, as well as of Cd levels in kidneys, were common features of Bulgarian (Markov et al. 2016) and Hungarian (present study) results.

The higher Pb concentration in liver samples found based on the full set of data is in accordance with the results of Bulgarian agricultural areas (Markov et al. 2016), while the lack of organ dependent significant differences in concentrations observed based on paired tests confirm the results of Georgiev et al. (2018).

To our knowledge, the current study performed the first comparisons of simultaneous concentrations of Al, Ca, Cr, Fe, K, Mg, Mn, and Na in golden jackal liver and kidney samples.

The potential explanations for our findings in jackals are discussed together with those noted in foxes.

In foxes, higher mean concentrations of Cu, Fe, Mn, Pb, and Zn occurred in livers compared to kidneys, but Ca and Na were higher in kidneys (Table S7).

Accumulations of Al, Cr, Mn, and Na in red fox liver and kidney samples had not been studied before. The majority of ecotoxicological studies performed on red fox kidney and liver samples focused on highly toxic, nonessential trace elements such as Cd and Pb or on elements from the group of most important and common essential metals associated with pollution: Cu, Fe, and Zn (Heltai and Markov 2012; Pérez-López et al. 2015; Binkowski et al. 2016; Georgiev et al. 2018; Zietara et al. 2019). Comparisons with these study results are a little cumbersome because their main aim was not to assess differences in hepatic and renal concentrations.

Taking the abovementioned limitations into consideration, the higher level of renal Cd in comparison with the hepatic Cd observed in our study appears to occur commonly in foxes in all the European habitats (Heltai and Markov 2012; Pérez-López et al. 2015; Binkowski et al. 2016; Georgiev et al. 2018; Zietara et al. 2019). Additionally, we detected this characteristic in jackals as well. This result is consistent with the one and only available comparison from Bulgaria (Markov et al. 2016).

Cadmium accumulates both in the livers and kidneys of terrestrial endothermic animals (Kalisińska 2019a). Furthermore, the mutual proportions between these organs suggest the nature of exposure (Tomza-Marciniak et al. 2019). It is widely accepted that the specific toxicokinetic of Cd bioaccumulation starts in the kidneys, and with further exposure, the concentration reaches a saturation level before the liver becomes the second most important organ of deposition (Herber 2004; Hernández-Moreno et al. 2013). Significantly higher Cd concentration in kidneys compared to livers generally suggests a lower level of exposure to this metal (Binkowski et al. 2016; Tomza-Marciniak et al. 2019).

Our finding concerning the higher level of Pb in fox livers than in fox kidneys appears to be consistent with other results ranging from Spain to Poland and Bulgaria (Pérez-López et al. 2015; Binkowski et al. 2016; Georgiev et al. 2018; Zietara et al. 2019) as well as with those noted in tissue samples of jackals from some Bulgarian agricultural areas (Markov et al. 2016). This latter result is also consistent with our findings based on the full set of data. However, there were no higher renal Pb levels in foxes in Hungary (Heltai and Markov 2012) and no evidenced (e.g., our results based on paired tests) or apparent, organ-dependent Pb accumulations in jackals from the Bulgarian mountains (Georgiev et al. 2018).

Though Pb accumulations tend to primarily target the nervous system, especially the brain, the highest concentrations usually occur in the bones (Kalisińska 2019a). However, studies performed on humans show that in addition to the nervous and skeletal systems, background levels of lead accumulate in concentrations of similar magnitude in almost all internal organs (Baranowska-Bosiacka et al. 2019). Also, in addition to Pb accumulations, excretion processes also occur through urine, feces, sweat, milk, and saliva (Baranowska-Bosiacka and Chlubek 2006). A relatively recent study performed on rats, but with implications on human health, proved that lead can be mobilized even from bones, which had once been considered permanent places of deposition (Conti et al. 2012). In this context, the renal and hepatic Pb levels seem to be in permanent flux depending on local habitat contamination. Therefore, biomonitoring studies should focus on differences among habitats and chronological fluctuations within certain habitats instead of on differences of concentrations among organs.

Our results showing higher concentration levels of Zn in liver samples are similar to those found in the majority of European study sites in both foxes (Heltai and Markov 2012; Pérez-López et al. 2015; Binkowski et al. 2016; Zietara et al. 2019) and jackals (Markov et al. 2016). The single exception was found in foxes collected from Bulgarian mountainous habitats (Georgiev et al. 2018) where the concentrations of Zn among liver and kidney samples were similar (liver, 30.106 mg/kg dw; kidney, 30.157 mg/kg dw).

In wild and domesticated canine species, the range of Zn concentration considered as optimal is slightly higher in the liver than in the kidneys (Kosik-Bogacka and Łanocha-Arendarczyk 2019). This suggests that our results, like those of the other studies in this topic, are convergent in terms of organ-related Zn concentration.

Differences between concentrations of renal and hepatic Cu found in foxes and in jackals were consistent among European study sites, concentration levels being higher in fox (Heltai and Markov 2012; Binkowski et al. 2016; Georgiev et al. 2018) and jackal livers as well (Markov et al. 2016).

Cu is present in every tissue of the bodies of mammals, but the liver is the main organ responsible for storing it (Osredkar and Sustar 2011). Toxic thresholds for Cu in predatory mammals were not found (Łanocha-Arendarczyk and Kosik-Bogacka 2019), but in a dog breed, hepatic copper storage was associated with hepatocellular damage and subclinical hepatitis (Mandigers et al. 2004). Therefore, Cu concentrations, at least in the livers of predatory mammals, should be a subject of the further ecotoxicological studies.

The higher hepatic Fe concentration found both in foxes and jackals is also in accordance with other available European study results, e.g., from Poland in foxes (Binkowski et al. 2016) or from Bulgaria in jackals (Markov et al. 2016).

Based on a comprehensive literature review performed by Kosik-Bogacka et al. (2019), the main areas of Fe accumulation in mammals are the liver and spleen, while kidneys, skeletal muscles, and bone marrow are secondary deposition places. Our results, together with those cited, strengthen this knowledge.

As far as we know, simultaneous concentration levels of renal and hepatic K in jackals had not been tested before. Our result shows no organ-dependent differences of K concentrations in jackals or foxes. Binkowski et al. (2016) attained similar results for foxes in Poland.

### Sex and age group effect

Sex-dependent differences in concentrations of selected elements were found in the kidney tissues of golden jackals in the cases of three elements (Al, Mn, and Zn) with higher mean values in males (Table S8). In jackal liver samples, Cu and Zn had higher sex-dependent concentrations in females (Table S10).

In jackals, sex-related analyses of concentration of chemical elements were previously performed only in a few cases (e.g., in Serbia and Romania) without any significant differences observed (Ćirović et al. 2015; Farkas et al. 2017). Other ecotoxicological studies considered only males (Markov et al. 2016) or did not mention the sex of the collected specimens (Georgiev et al. 2018).

We found no sex-related differences in accumulations of selected elements in kidneys or in livers of foxes (Table S9). These results are in accordance with those noted in liver samples from Romania (36 males and 20 females) where only Mn concentration had higher values in males (Farkas et al. 2017). Similarly, significant differences between sexes were not found in hepatic and renal concentrations of Cd, Pb, and Zn in foxes from Spain (Pérez-López et al. 2015) nor of Cd, Fe, Hg, K, Mg, Pb, and Zn from Poland (Binkowski et al. 2016). However, the Cu concentrations in liver samples of foxes (3 females and 13 males) were similar among sexes in Spain (Millán et al. 2008), but in kidneys from Poland (8 females and 6 males), higher values were discovered in females (Binkowski et al. 2016). Also, the Pb concentration presented higher values in females from highly polluted areas in Poland as well as from protected natural areas within the Doñana National and Natural Parks, Spain (Millán et al. 2008; Zietara et al. 2019). Interestingly, in the same habitats, Cd had higher concentrations in male foxes from Spain. Conversely, in Poland, tissue samples collected from females had higher levels of Cd (Millán et al. 2008; Zietara et al. 2019). The Zn accumulation seems to be constant between sexes among all referred study sites (Pérez-López et al. 2015; Binkowski et al. 2016; Zietara et al. 2019).

Burger (2007) recommends that authors clearly describe sex differences when examining the accumulation of certain metals or other contaminants, and that those authors should state if they had the possibility to identify such differences. She suggests there are many individual features (i.e., size, nutrition, genetics, and hormones) that could reflect as sex-related differences in exposure and susceptibility. However, within- or close to our study site, investigations about trophic relationships between the golden jackal and red fox revealed similar feeding habits (Lanszki and Heltai 2002; Lanszki et al. 2006; Lanszki et al. 2016) without relevant detectable differences between the sexes (Lanszki et al. 2015). In this context, similar feeding habits could explain the lack of sex-related differences in accumulations of the selected elements. Reproductive status could also explain some patterns of sex-dependent prevalence of certain elements (Vahter et al. 2007). Nevertheless, the sample collection method works against this kind of investigation because regular hunting is generally more intensive during the winter, which is a period of decreased sexual activity. Another limitation of sex-related examinations could be the sample size within a study site. When we compared our results with reference works during the interpretation process, we observed that the sex-related differences dwindled as the number of samples increased. Zietara et al. (2019) may offer a simpler explanation. Their study suggested that the differences between genders may remain hidden at low environmental exposure levels. In order to fulfill the recommendations of Burger (2007), future ecotoxicological studies that test sex differences should consider the sample size as well as the sample collection period.

Age group-related differences were found in Cu and K concentrations, with higher values in the liver samples of juvenile jackals (Table S10). Our results can only be compared with those discovered in southern Romania (Farkas et al. 2017), where age group-related differences in jackal liver samples were similarly not detected. In other European study sites (e.g., Serbia and Bulgaria), either only adult specimens were collected (Ćirović et al. 2015; Markov et al. 2016) or the age group-dependent accumulations were not considered (Georgiev et al. 2018).

We could not perform age group-related comparisons in foxes due to a lack of juvenile specimens. However, such kinds of investigations are more frequent for foxes than for jackals. In most of the study sites (e.g., southern Romania, the Lower Silesian forest, or Małopolska Province, Poland), concentrations of the majority of the selected elements showed no correlation with the age of foxes (Binkowski et al. 2016; Farkas et al. 2017; Zietara et al. 2019). Available study results without significant age group-related differences in kidney samples do exist at the level of some particular elements of higher importance, such as Pb (Dip et al. 2001; Pérez-López et al. 2015). The same holds true for higher concentrations in livers of adults (Pérez-López et al. 2015) or conversely, higher levels of residues in juvenile ages in kidney samples from Polish forested habitats and in liver samples from Swiss urban areas (Dip et al. 2001; Binkowski et al. 2016). Cadmium concentrations appear to be more consistent among European study sites: both hepatic and renal concentrations were higher in adults than in juveniles in north-western Spain (Pérez-López et al. 2015), similar to residues found in liver samples from southern Spain (Millán et al. 2008) or suburban and urban areas from Switzerland (Dip et al. 2001). The Zn accumulation displayed no significant differences between age groups among urban, suburban, and rural areas (Dip et al. 2001), or both hepatic and renal concentrations were higher in juveniles (Pérez-López et al. 2015).

Although the study results of Pérez-López et al. (2015) argue that age is an important parameter to include in biomonitoring programs focusing on trace metal bioaccumulation in red foxes, this factor seems to be controversial in a broader context.

### Element concentrations among European study sites

#### Al, the third most abundant element in nature

There are no maximum tolerated levels (MTLs) of aluminum in free-living mammals. A value has been set for rodents of 200 mg kg^−1^ dry weight, and a five times higher level is considered acceptable for farm animals (Skibniewska and Skibniewski 2019). The average concentrations found in jackal livers and kidneys, as well as in fox livers, are far below these values. However, Al seems to be a nonessential chemical element without large interest in ecotoxicological studies performed on mesopredators. The one and single reference found is from Romania with similar mean concentration in livers of jackals and foxes (Farkas et al. 2017).

#### Essential macroelements or mineral nutrients: Ca, K, Mg, and Na

As Davydov et al. (2020) stated: “the availability of sufficient amounts of mineral nutrition is one of the fundamental factors required for survival and growth of large herbivore populations.” As essential elements engage in the metabolic processes of all living organisms, an adequate mineral concentration (above deficiency symptoms) could be desirable in carnivores as well. Among European study sites, the highest Ca concentration was found in the liver samples of jackals and foxes from Romania (Farkas et al. 2017), while the lowest values come from Polish foxes (Binkowski et al. 2016). We found no values or terms of comparison for jackal kidney samples in the literature; however, with jackal livers, our mean concentration data was approximately 6 times lower than recorded concentration data from Romania (Farkas et al. 2017). Renal concentrations in our foxes were the highest among literature data, but liver concentrations were between the lowest (Polish) and highest (Romanian) mean values (Table 3).

**Table 3:** Concentration levels of selected elements among European study sites (mg/kg dw)

Country	Organ	Al	Ca	Cd	Cr	Cu	Fe	K	Mg	Mn	Na	Pb	Zn	Source
Golden jackal														
Hungary	Liver	19.27	202	0.19	0.45	58.74	718.6	8123.84	488.79	11.35	3392.14	5.53	110.65	Present study
Hungary	Kidney	13.82	285.36	0.44	0.32	14.62	257.58	7941.74	550.14	5.2	5425.29	3.44	74.08	Present study
Serbia	Liver			14.89		57.85	1017.27			16.93		9.59	66.36	Ćirović et al. (2015)
Romania	Liver	12.67	1189.31	0.29	0.48	79.65	669.75		883.05	13.8		1.29		Farkas et al. (2017)
Bulgaria	Liver			0.58		57.62						6.88	141.45	Markov et al. (2016)
Bulgaria	Kidney			1.41		17.67						4.03	58.28	Markov et al. (2016)
Bulgaria	Liver			11.57		56.32						8.88	63.62	Georgiev et al. (2018)
Bulgaria	Kidney			11.19		33.86						8.42	61.58	Georgiev et al. (2018)
Red fox														
Hungary	Liver	20.54	247.39	0.33	0.51	40.86	585.5	7898.5	503.63	10.42	3872.34	7.41	112.23	Present study
Hungary	Kidney	15.72	298.55	0.93	0.39	11.87	194.83	8499.77	540.44	4.58	5525.14	3.74	65.96	Present study
Romania	Liver	18.41	1333.37	3.51	1.37	63.47	746.03		952.81	15.9		1.88		Farkas et al. (2017)
Poland	Liver			7.46								7.43	130.64	Zietara et al. (2019)
Poland	Kidney			22.93								2.26	73.73	Zietara et al. (2019)
Poland	Liver		18.15	1.29		30.39	372.31	10092.31	734.91			1.66	128.26	Binkowski et al. (2016)
Poland	Kidney		10.15	1.66		13.3	176.91	10481.3	538.15			1.64	58.58	Binkowski et al. (2016)
Spain	Liver			0.58								0.81	77	Pérez-López et al. (2015)
Spain	Kidney			1.28								0.06	17	Pérez-López et al. (2015)
Hungary	Liver			0.50		21.42						1.68	156.93	Heltai and Markov (2012)
Hungary	Kidney			0.82		9.24						2.63	87.16	Heltai and Markov (2012)
Bulgaria	Liver			0.63		15.12						0.95	30.11	Georgiev et al. (2018)
Bulgaria	Kidney			15.52		8.47						0.76	30.16	Georgiev et al. (2018)
Spain	Liver			0.12		72						0.26	136.7	Millán et al. (2008)

Potassium (K) concentrations in mesocarnivores were investigated only by Binkowski et al. (2016) who found values around 10,000 mg kg^−1^ dw in fox livers and kidneys. Our results indicate balanced mean K concentrations as well, but the values are around 8000 mg kg^−1^ dw among studied species and organs.

Magnesium (Mg) concentrations in liver samples were the lowest among European study sites. Renal Mg concentrations observed by Binkowski et al. (2016) in Poland are similar to our results.

Our results concerning the Na concentrations in jackals and foxes are the first values in this study area.

#### Heavy metals

From the selected elements, Cd, Cr, Cu, Fe, Mn, Pb, and Zn cumulatively fulfill the criteria for heavy metals delineated by Ali and Khan (2018). These criteria include natural occurrence, greater atomic number (Z) than 20, and an elemental density greater than 5 g cm^−3^. The biological statuses of some of those elements (i.e., Pb and Cd) are considered nonessential and toxic, while others (Cr, Cu, Fe, and Mn) are well known as essential or probably essential (e.g., Mn) elements (WHO 1996; Tchounwou et al. 2012; Roth et al. 2013; Ali et al. 2019; Kalisińska 2019a). The biological status of Zn is controversial. Some sources regard the element as essential to the growth and development of organisms (WHO 1996), while others refer to it as nonessential (Kalisińska 2019a). Nonessential heavy metals may be toxic even at low concentrations, while essential heavy metals are required in trace quantities but become toxic beyond certain limits or threshold concentrations (Ali et al. 2019). The normal and toxic threshold values of element concentrations as well as deficiency levels for essential elements are readily available (Table S11). In the light of threshold concentrations, most toxic Pb concentrations observed at our study site fall within the normal range among tested organs and species. Cd, the second element associated with environmental pollution, is also present in trace quantities in kidneys of jackals and foxes in comparison with toxic levels. Concentrations of the remaining selected elements, those that are biologically essential or probably essential (Cu, Fe, Mn, and Zn) are far below the toxic levels in both organs of the examined species, with a slight sign of deficiency of Fe, Mn, and Zn in the kidneys of jackals and foxes as well. In liver samples, concentrations of essential elements were within normal ranges both in jackals and foxes.

**Fig. 2:**
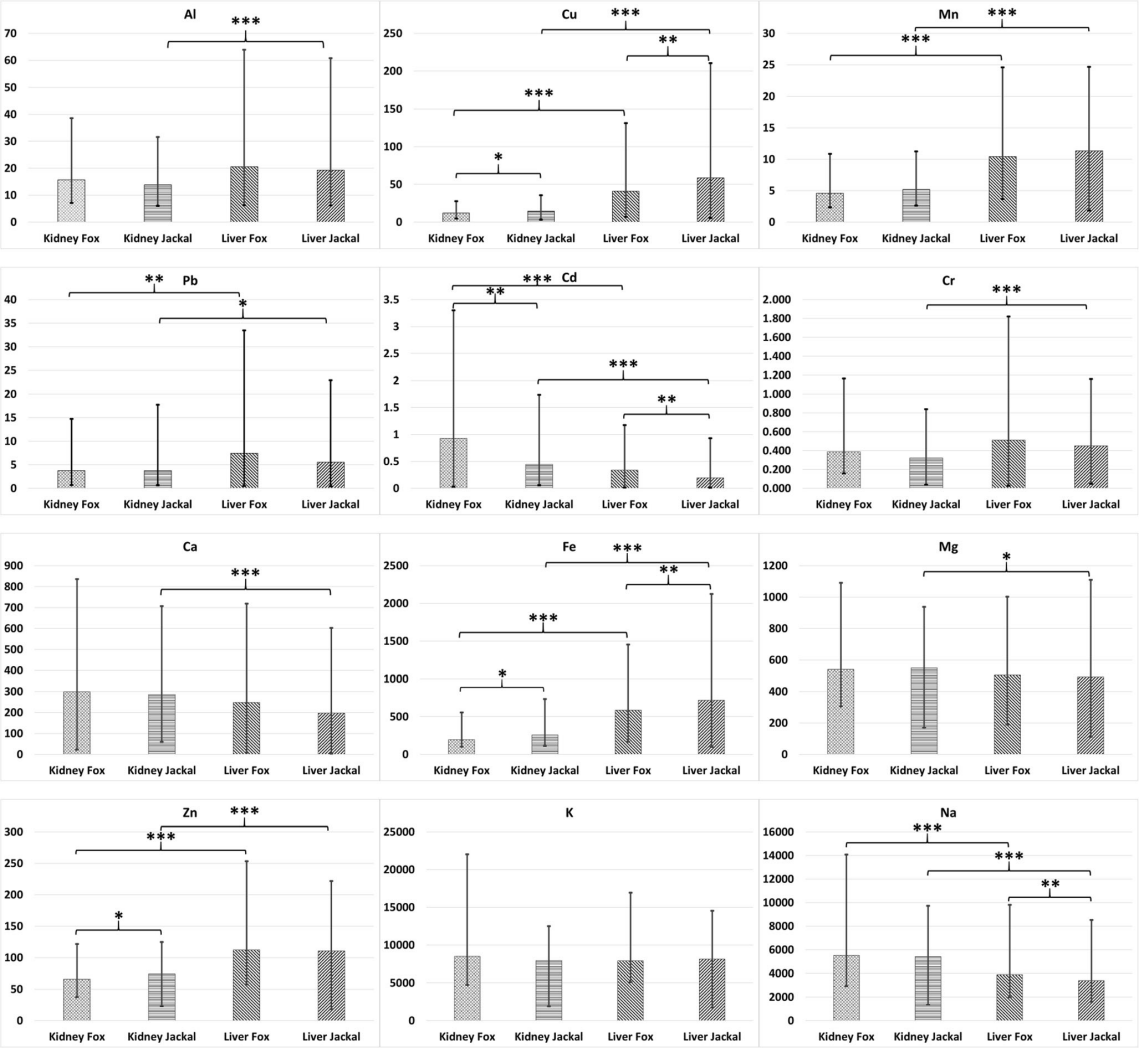
Differences in concentrations of selected elements (mg/kg dw) between species (Red fox vs. Golden jackal) and organs (kidney vs. liver). Mean, minimum and maximum values are marked in box whisker plots. Between-species differences were tested with T-test for independent samples by group and Mann-Whitney U Test; differences among the organs were tested with T-test for independent samples assuming equal variances and T-test for independent samples with separate variance estimates; *p < 0.05, **p < 0.01, ***p < 0.001

In terms of Cd contamination, our study site could be considered as one of the least polluted in Europe (Table 3) since we found the lowest concentrations both in liver and kidney samples of jackals as well as in those of foxes, with the exception of the liver results of foxes from southern Spain (Millán et al. 2008). Lead (Pb) concentrations in jackal liver samples fall in the range of 5.53–9.59 mg kg^−1^ dw (Ćirović et al. 2015; Markov et al. 2016; Farkas et al. 2017; Georgiev et al. 2018) including our study results with the lowest mean values. In jackal kidney samples, lower mean concentrations than those in our study were found only in Romania (Farkas et al. 2017). In fox liver samples, we uncovered the second highest mean Pb concentrations (in absolute values but similar as magnitude) after those found in Małopolska Province, Poland (Zietara et al. 2019), while our kidney sample values are the highest among all European study sites. The source of these high Pb concentrations needs to be investigated. Our Cr concentrations are similar in jackal livers and lower in fox livers when compared to values from Romania (Farkas et al. 2017). Concerning kidney samples, it seems that we set the first reference data for Cr concentrations for jackals and foxes as well. Higher mean Cu concentrations in jackal liver samples occurred only in Romania (Farkas et al. 2017), but the average values were similar among all study sites (Ćirović et al. 2015; Markov et al. 2016; Georgiev et al. 2018). In fox liver samples, our Cu concentration results were the third highest after those found in Spain (Millán et al. 2008) and Romania (Farkas et al. 2017). Our study contained the second highest Cu concentrations after values from the Lower Silesian forest, Poland (Binkowski et al. 2016). Iron (Fe) concentrations in jackal liver samples are slightly similar to those found in Romania (Farkas et al. 2017), but much lower than data from Serbia (Ćirović et al. 2015). Our study set the first reference Fe concentration value for golden jackal kidney samples. The concentration levels our study observed in fox livers were between the lowest from Poland (Binkowski et al. 2016) and highest from Romania (Farkas et al. 2017). In kidney samples, our Fe concentrations were higher than in the single European study site from Poland (Binkowski et al. 2016). Manganese (Mn) concentrations were the lowest among jackal and fox liver samples, while with kidney samples, we set the first reference values for both species. Our Zn concentrations were the second highest among jackal liver and kidney samples. In fox liver samples, the Zn concentrations ranged between 30 and 160 mg kg^−1^ dw. Hepatic Zn concentrations were above 110 mg kg^−1^ dw in five out of seven European study sites (Fig. 2). Our result falls within this range. The concentration ranges are narrower in kidney samples, between 17 and 87.16 mg Zn kg^−1^ dw. Our results are closer to the upper values and are ranked third highest behind study sites from Hungary (Heltai and Markov 2012) and Poland (Zietara et al. 2019).

## Conclusions

The sympatric golden jackals and red foxes from the selected habitat are suitable for ecotoxicological studies because all 12 elements were detected in various concentrations independently of the organ tested (kidney or liver). As a result of sympatry and simultaneous sample collection, the intraspecific differences may only be sought in potentially differing metabolisms or diets.

Our results demonstrate that the detection of the differing concentrations of certain elements among liver and kidney samples in jackals could depend on testing methods (random sampling vs. data pairs) or sample sizes.

Because we discovered no sex-dependent difference in concentrations of selected elements in red fox kidneys or livers, and this kind of differences occurred in jackals only in three elements out of 12, we suggest that studies concerning concentrations of chemical residuals in organ samples of mesocarnivores should consider the sex effect only in relation with reproductive status in the sample collection period.

Our age group-related investigations of selected element concentrations do not confirm possible processes of bioaccumulation; therefore, kidney and liver samples collected from jackals and foxes belonging to different age groups can be used mixed in ecotoxicological studies.

As concentrations of nonessential elements as well as those of concentration-dependent toxicity fall within values accepted as the limits of normal ranges, the sample collection area of Somogy County in Hungary can be considered one of the areas least exposed to environmental pollution among the European study sites.

## Supplementary Information

ESM 1 (DOCX 80 kb)

## Data Availability

The datasets used and analyzed during the current study are available from the corresponding author on reasonable request.

## References

[CR1] Ali H, Khan E (2018). What are heavy metals? Long-standing controversy over the scientific use of the term “heavy metals” – proposal of a comprehensive definition. Toxicol Environ Chem.

[CR2] Ali H, Khan E, Ilahi I (2019). Environmental chemistry and ecotoxicology of hazardous heavy metals: environmental persistence, toxicity, and bioaccumulation. J Chem-NY.

[CR3] Banea OC, Farkas A, Stoyanov S et al (2018) Red fox and golden jackal hunting bag differences in countries from Central and Southeastern Europe. Population trend and management aspects. In: Giannatos G, Banea OC, Hautlauf J et al (eds) Proceedings of the 2nd International Symposium on jackals and related species. Hellenic Zoological Society, Marathon Bay - Attica, pp 121–122

[CR4] Baranowska-Bosiacka I, Chlubek D (2006). Biochemical mechanisms of neurotoxic lead activity. Postepy Biochem.

[CR5] Baranowska-Bosiacka I, Korbecki J, Marchlewicz M (2019) Lead, Pb. In: Kalisińska E (ed) Mammals and birds as bioindicators of trace element contaminations in terrestrial environments. Springer Nature, Switzerland, pp 563–592

[CR6] Binkowski ŁJ, Merta D, Przystupińska A, Sołtysiak Z, Pacoń J, Stawarz R (2016). Levels of metals in kidney, liver and muscle tissue and their relation to the occurrence of parasites in the red fox in the Lower Silesian forest in Europe. Chemosphere.

[CR7] Burger J (2007). A framework and methods for incorporating gender-related issues in wildlife risk assessment: gender-related differences in metal levels and other contaminants as a case study. Environ Res.

[CR8] Ćirović D, Gizejewska A, Jovanović V (2015). Concentration of selected trace elements in the golden jackal (Canis aureus l., 1758) population from Serbia. Acta Zool Bulgar.

[CR9] Conti MI, Terrizzi AR, Lee CM, Mandalunis PM, Bozzini C, Piñeiro AE, Martínez MP (2012). Effects of lead exposure on growth and bone biology in growing rats exposed to simulated high altitude. Bull Environ Contam Toxicol.

[CR10] Csányi S, Márton M, Kovács V et al (2018) Hungarian game management database 2017/2018. Szent István Egyetem, Gödöllő

[CR11] Davydov S, Davydova A, Schelchkova M, Makarevich R, Fyodorov-Davydov D, Loranty M, Boeskorov G (2020). Essential mineral nutrients of the high-latitude steppe vegetation and the herbivores of mammoth fauna. Quat Sci Rev.

[CR12] Dip R, Stieger C, Deplazes P (2001). Comparison of heavy metal concentrations in tissues of red foxes from adjacent urban, suburban, and rural areas. Arch Environ Contam Toxicol.

[CR13] Farkas A, Bidló A, Bolodár-Varga B, Jánoska F (2017). Accumulation of metals in liver tissues of sympatric golden jackal (Canis aureus) and red fox (Vulpes vulpes) in the Southern part of Romania. Bull Environ Contam Toxicol.

[CR14] Georgiev D, Raichev E, Dospatliev L (2018). Heavy metals concentrations in organs of red foxes (Vulpes vulpes Linnaeus, 1758) and golden jackals (Canis aureus Linnaeus, 1758) inhabiting the “Sarnena Sredna gora.”. Bulg J Agric Sci.

[CR15] Heltai M, Markov G (2012). Red fox (Vulpes vulpes Linnaeus, 1758) as biological indicator for environmental pollution in Hungary. Bull Environ Contam Toxicol.

[CR16] Herber R (2004) Cadmium. In: Merian E, Anke M, Ihnat M, Stoeppler M (eds) Elements and their compounds in the environment. WILEY-VCH Verlag GMBH & Co. KGaA, Weinheim, pp 689–708

[CR17] Hernández-Moreno D, De la Casa RI, Fidalgo LE (2013). Noninvasive heavy metal pollution assessment by means of Iberian wolf (Canis lupus signatus) hair from Galicia (NW Spain): a comparison with invasive samples. Environ Monit Assess.

[CR18] Kalisińska E (2019a) Endothermic animals as biomonitors of terrestrial environments. In: Kalisińska E (ed) Mammals and birds as bioindicators of trace element contaminations in terrestrial environments, vol 2019. Springer Nature, Switzerland, pp 21–53

[CR19] Kalisińska E (2019b) Human population increase and changes in production and usage of trace elements in the twentieth century and first decades of the twenty-first. In: Mammals and birds as bioindicators of trace element contaminations in terrestrial environments. Springer Nature, Switzerland, pp 3–20

[CR20] Keresztesi Á, Birsan MV, Nita IA, Bodor Z, Szép R (2019) Assessing the neutralisation, wet deposition and sourcecontributions of the precipitation chemistry over Europe during 2000 – 2017. Environ Sci Eur 31:1–15. 10.1186/s12302-019-0234-9

[CR21] Keresztesi Á, Nita I, Birsan MV, Bodor Z, Szép R (2020) The risk of cross-border pollution and the influence of regional climate on the rainwater chemistry in the Southern Carpathians , Romania. Environ Sci Pollut Res 27:9382–9402. 10.1007/s11356-019-07478-9 10.1007/s11356-019-07478-9 PMC7089915 31916162

[CR22] Kosik-Bogacka DI, Łanocha-Arendarczyk N (2019) Zinc, Zn. In: Kalisińska E (ed) Mammals and birds as bioindicators of trace element contaminations in terrestrial environments. Springer Nature Switzerland AG, Berlin, pp 363–411

[CR23] Kosik-Bogacka DI, Łanocha-Arendarczyk N, Kalisińska E et al (2019) Iron, Fe. In: Kalisińska E (ed) Mammals and birds as bioindicators of trace element contaminations in terrestrial environments. Springer Nature Switzerland AG, Berlin, pp 181–212

[CR24] Łanocha-Arendarczyk N, Kosik-Bogacka DI (2019) Copper, Cu. In: Kalisińska E (ed) Mammals and birds as bioindicators of trace element contaminations in terrestrial environments. Springer Nature Switzerland AG, Berlin, pp 125–161

[CR25] Lanszki J, Heltai M (2002). Feeding habits of golden jackal and red fox in south-western Hungary during winter and spring. Mamm Biol.

[CR26] Lanszki J, Heltai M, Szabo L (2006). Feeding habits and trophic niche overlap between sympatric golden jackal (Canis aureus) and red fox (Vulpes vulpes) in the Pannonian ecoregion (Hungary). Can J Zool.

[CR27] Lanszki J, Kurys A, Heltai M, Csányi S, Ács K (2015). Diet composition of the golden jackal in an area of intensive big game management. Ann Zool Fenn.

[CR28] Lanszki J, Kurys A, Szabó L (2016). Diet composition of the golden jackal and the sympatric red fox in an agricultural area (Hungary). Folia Zool Brno.

[CR29] Mandigers PJJ, van den Ingh TSGAM, Bode P (2004). Association between liver copper concentration and subclinical hepatitis in Doberman Pinschers. J Vet Intern Med.

[CR30] Markov G, Kocheva M, Gospodinova M (2016). Assessment of heavy metal accumulation in the golden jackal (Canis aureus) as a possible bioindicator in an agricultural environment in Bulgaria. Bull Environ Contam Toxicol.

[CR31] Millán J, Mateo R, Taggart MA, López-Bao JV, Viota M, Monsalve L, Camarero PR, Blázquez E, Jiménez B (2008). Levels of heavy metals and metalloids in critically endangered Iberian lynx and other wild carnivores from Southern Spain. Sci Total Environ.

[CR32] Nagy G, Ács K, Csivincsik Á (2014). The occurrence of thorny-headed worm Macracanthorhynchus hirudinaceus in Transdanubian wild boar populations in relation to certain environmental factors. Bull Forest Sci.

[CR33] Nordberg GF, Fowler BA, Nordberg M (2015) Toxicology of metals: overwiew, definitions, concepts, and trends. In: Nordberg GF, Fowler BA, Nordberg M (eds) Handbook of the toxicology of metals, Fourth edi. Academic Press an imprint of Elsevier

[CR34] Osredkar J, Sustar N (2011) Copper and zinc, biological role and significance of copper / zinc imbalance. J Clin Toxicol s3. 10.4172/2161-0495.S3-001

[CR35] Pérez-López M, Rodríguez FS, Hernández-Moreno D, Rigueira L, Fidalgo LE, Beceiro AL (2015). Bioaccumulation of cadmium, lead and zinc in liver and kidney of red fox (Vulpes vulpes) from NW Spain: influence of gender and age. Toxicol Environ Chem.

[CR36] Pohl P, Kalinka M, Pieprz M (2019). Development of a very simple and fast analytical methodology for FAAS/ FAES measurements of Ca, K, Mg and Na in red beetroot juices along with chemical fractionation of Ca and Mg by solid phase extraction. Microchem J.

[CR37] Reimann C, Fabian K, Birke M, Filzmoser P, Demetriades A, Négrel P, Oorts K, Matschullat J, de Caritat P, Albanese S, Anderson M, Baritz R, Batista MJ, Bel-Ian A, Cicchella D, de Vivo B, de Vos W, Dinelli E, Ďuriš M, Dusza-Dobek A, Eggen OA, Eklund M, Ernsten V, Flight DMA, Forrester S, Fügedi U, Gilucis A, Gosar M, Gregorauskiene V, de Groot W, Gulan A, Halamić J, Haslinger E, Hayoz P, Hoogewerff J, Hrvatovic H, Husnjak S, Jähne-Klingberg F, Janik L, Jordan G, Kaminari M, Kirby J, Klos V, Kwećko P, Kuti L, Ladenberger A, Lima A, Locutura J, Lucivjansky P, Mann A, Mackovych D, McLaughlin M, Malyuk BI, Maquil R, Meuli RG, Mol G, O'Connor P, Ottesen RT, Pasnieczna A, Petersell V, Pfleiderer S, Poňavič M, Prazeres C, Radusinović S, Rauch U, Salpeteur I, Scanlon R, Schedl A, Scheib A, Schoeters I, Šefčik P, Sellersjö E, Slaninka I, Soriano-Disla JM, Šorša A, Svrkota R, Stafilov T, Tarvainen T, Tendavilov V, Valera P, Verougstraete V, Vidojević D, Zissimos A, Zomeni Z, Sadeghi M (2018). GEMAS: establishing geochemical background and threshold for 53 chemical elements in European agricultural soil. Appl Geochem.

[CR38] Roth J, Ponzoni S, Aschner M (2013) Manganese homeostasis and transport. In: Banci L (ed) Metallomics and the cell, metal ions in life sciences. Springer Netherlands, pp 169–201 10.1007/978-94-007-5561-1_6 PMC6542352 23595673

[CR39] Sedláková J, Řezáč P, Fišer V, Hedbávný J (2019). Red Fox, Vulpes vulpes L., as a bioindicator of environmental pollution in the countryside of Czech Republic. Acta Univ Agric Fac Agron.

[CR40] Skibniewska E, Skibniewski M (2019) Aluminum, Al. In: Kalisińska E (ed) Mammals and birds as bioindicators of trace element contaminations in terrestrial environments, vol 2019. Springer Nature Switzerland AG, pp 413–462

[CR41] Tchounwou PB, Yedjou CG, Patlolla AK, Sutton DJ (2012) Heavy metal toxicity and the environment. EXS:133–164. 10.1007/978-3-7643-8340-4_6 10.1007/978-3-7643-8340-4_6 PMC4144270 22945569

[CR42] Tomza-Marciniak A, Pilarczyk B, Marciniak A et al (2019) Cadmium, Cd. In: Kalisińska E (ed) Mammals and birds as bioindicators of trace element contaminations in terrestrial environments. Springer Nature Switzerland AG, Berlin, pp 483–532

[CR43] Vahter M, Agneta A, Liden C (2007). Gender differences in the disposition and toxicity of metals. Environ Res.

[CR44] WHO (1996) Trace elements in human nutrition and health. In: World Health Organization. WHO Press, Geneva

[CR45] Zietara J, A. Wierzbowska I, Gdula-Argasinska J (2019). Concentrations of cadmium and lead, but not zinc, are higher in red fox tissues than in rodents — pollution gradient study in the Małopolska province (Poland). Environ Sci Pollut Res.

