# Supplementary material for: Accumulation of selected metals and concentration of macroelements in liver and kidney tissues of sympatric golden jackal (Canis aureus) and red fox (Vulpes vulpes) in Somogy County, Hungary
Source: Environ Sci Pollut Res Int. 2021 Jul 8;28(47):66724–35. doi: 10.1007/s11356-021-15156-y (PMC8642344; doi:10.1007/s11356-021-15156-y)
Supplement: Supplementary file 1 — (DOCX 80 kb) [file 11356_2021_15156_MOESM1_ESM.docx]

**Supplementary materials for:**

**Accumulation of selected metals and concentration of macroelements in liver and kidney tissues of sympatric golden jackal (Canis aureus) and red fox (Vulpes vulpes) in Somogy County, Hungary**

Farkas, Attila^1^, Bidló, András^2^, Bolodár-Varga, Bernadett^2^, Jánoska, Ferenc^3^

1: Sapientia Hungarian University of Transilvania, Faculty of Technical and Human Sciences, Târgu-Mureș, 540485 Târgu-Mureş, Corunca, 1C, Romania, e-mail: [farkas.attila@ms.sapientia.ro](mailto:farkas.attila@ms.sapientia.ro)

2: University of Sopron, Faculty of Forestry, [Institute of Environmental and Earth Sciences](http://www.emk.nyme.hu/index.php/14454/), H-9400 Sopron, Bajcsy-Zs. str. 4, Hungary, e-mail: [bidlo.andras@uni-sopron.hu](mailto:bidlo.andras@uni-sopron.hu); [varga.bernadett@uni-sopron.hu](mailto:varga.bernadett@uni-sopron.hu)

3: University of Sopron, Faculty of Forestry, Institute of Wildlife Management and Vertebrate Zoology, H-9400 Sopron, Bajcsy-Zs. str. 4, Hungary, e-mail: [janoska.ferenc@uni-sopron.hu](mailto:janoska.ferenc@uni-sopron.hu)

Corresponding author: Farkas Attila, e-mail: [farkas.attila@ms.sapientia.ro](mailto:farkas.attila@ms.sapientia.ro), tel.: +40 740 128 593; orcid.org/0000-0002-8995-557X

Table S1 – Number of total studied samples, of samples below detection limits (BDL), extreme values and valid samples

| Element | Kidney | | | | Liver | | | |
| --- | --- | --- | --- | --- | --- | --- | --- | --- |
|  | Total | BDL | Extreme | Valid | Total | BDL | Extreme | Valid |
| Al | 73 | 6 | 2 | 65 | 212 | 7 | 15 | 190 |
| Ca | 73 | 1 | 1 | 71 | 212 | 46 | 6 | 160 |
| Cd | 73 | 2 | 1 | 70 | 212 | 33 | 1 | 178 |
| Cr | 73 | 2 | 1 | 70 | 212 | 1 | 14 | 197 |
| Cu | 73 | 0 | 0 | 73 | 212 | 0 | 3 | 209 |
| Fe | 73 | 0 | 1 | 72 | 212 | 0 | 0 | 212 |
| K | 73 | 0 | 0 | 73 | 212 | 0 | 0 | 212 |
| Mg | 73 | 0 | 1 | 72 | 212 | 1 | 0 | 211 |
| Mn | 73 | 0 | 1 | 72 | 212 | 0 | 3 | 209 |
| Na | 73 | 0 | 1 | 72 | 212 | 0 | 2 | 210 |
| Pb | 73 | 0 | 6 | 67 | 212 | 0 | 44 | 168 |
| Zn | 73 | 0 | 1 | 72 | 212 | 0 | 1 | 211 |

*^BDL^* ^values below detection limits,^ *^Extreme^* ^numbers of data deemed to be extreme values when exceeds ± 3SD^

Table S2 - Concentrations of 12 selected elements depending on species, organs, sex, and age group

| Element | Jackal vs. Fox | | Kidney vs. Liver | | | | Male vs. Female | | | | Adult vs. Juvenile |
| --- | --- | --- | --- | --- | --- | --- | --- | --- | --- | --- | --- |
|  |  |  | Full data set | | Data pairs | | Jackal | | Fox | | Jackal |
|  | Kidney | Liver | Jackal | Fox | Jackal | Fox | Kidney | Liver | Kidney | Liver | Liver |
| Al | n.s. | n.s. | <0.001 | n.s. | n.s. | n.s. | **<0.05** | n.s. | n.s. | n.s. | n.s. |
| Ca | n.s. | n.s. | <0.01 | n.s. | n.s. | n.s. | n.s. | n.s. | n.s. | n.s. | n.s. |
| Cd | **<0.01** | **<0.001** | <0.001 | <0.001 | <0.001 | <0.001 | n.s. | n.s. | n.s. | n.s. | n.s. |
| Cr | n.s. | n.s. | <0.001 | n.s. | n.s. | n.s. | n.s. | n.s. | n.s. | n.s. | n.s. |
| Cu | <0.05 | <0.01 | **<0.001** | **<0.001** | **<0.001** | **<0.001** | n.s. | <0.01 | n.s. | n.s. | <0.01 |
| Fe | <0.05 | <0.01 | **<0.001** | **<0.001** | **<0.001** | **<0.001** | n.s. | n.s. | n.s. | n.s. | n.s. |
| K | n.s. | n.s. | n.s. | n.s. | n.s. | n.s. | n.s. | n.s. | n.s. | n.s. | <0.05 |
| Mg | n.s. | n.s. | <0.05 | n.s. | n.s. | n.s. | n.s. | n.s. | n.s. | n.s. | n.s. |
| Mn | n.s. | n.s. | **<0.001** | **<0.001** | **<0.001** | **<0.001** | **0.05** | n.s. | n.s. | n.s. | n.s. |
| Na | n.s. | **<0.01** | <0.001 | <0.001 | <0.001 | <0.001 | n.s. | n.s. | n.s. | n.s. | n.s. |
| Pb | n.s. | n.s. | <0.05 | **<0.01** | n.s. | **<0.05** | n.s. | n.s. | n.s. | n.s. | n.s. |
| Zn | <0.05 | n.s. | **<0.001** | **<0.001** | **<0.001** | **<0.001** | **<0.05** | <0.05 | n.s. | n.s. | n.s. |
| **^Note:^** ^Higher mean values highlighted according to the grouping variables: G1 – bold; G2 - underline^ | | | | | | | | | | | |
| **^G1^**  **^G2^** | **^Fox^**^;^  ^Jackal^ | | **^Liver;^**  ^Kidney^ | | | | **^Male;^**  ^Female^ | | | | **^Adult^**^;^  ^Juvenile^ |

Table S3 – Normality of variances for metals in kidney and liver samples of red foxes (Kolmogorov-Smirnov Tests)

| Element and tissue | Adult male | K-S test | | Adult female | K-S test | |
| --- | --- | --- | --- | --- | --- | --- |
|  | N | d-value | p-value | N | d-value | p-value |

| Al_K | 12 | 0.212 | > .20 | 12 | 0.231 | > .20 |
| --- | --- | --- | --- | --- | --- | --- |
| Ca_K | 13 | 0.250 | > .20 | 14 | 0.169 | > .20 |
| Cd_K | 12 | 0.189 | > .20 | 13 | 0.171 | > .20 |
| Cr_K | 12 | 0.272 | > .20 | 12 | 0.138 | > .20 |
| Cu_K | 13 | 0.148 | > .20 | 14 | 0.168 | > .20 |
| Fe_K | 13 | 0.209 | > .20 | 13 | 0.210 | > .20 |
| K_K | 13 | 0.195 | > .20 | 14 | 0.241 | > .20 |
| Mg_K | 13 | 0.138 | > .20 | 13 | 0.078 | > .20 |
| Mn_K | 13 | 0.170 | > .20 | 14 | 0.233 | > .20 |
| Na_K | 13 | 0.132 | > .20 | 14 | 0.164 | > .20 |
| Pb_K | 12 | 0.264 | > .20 | 14 | 0.146 | > .20 |
| Zn_K | 13 | 0.235 | > .20 | 13 | 0.173 | > .20 |
| Al_L | 29 | 0.227 | < .10 | 24 | 0.173 | > .20 |
| Ca_L | 29 | 0.192 | < .20 | 22 | 0.186 | > .20 |
| Cd_L | 30 | 0.173 | > .20 | 23 | 0.144 | > .20 |
| Cr_L | 29 | 0.182 | > .20 | 25 | 0.196 | > .20 |
| Cu_L | 31 | 0.190 | < .15 | 25 | 0.171 | > .20 |
| Fe_L | 32 | 0.127 | > .20 | 26 | 0.149 | > .20 |
| K_L | 32 | 0.096 | > .20 | 26 | 0.087 | > .20 |
| Mg_L | 32 | 0.109 | > .20 | 26 | 0.149 | > .20 |
| Mn_L | 32 | 0.135 | > .20 | 26 | 0.073 | > .20 |
| Na_L | 32 | 0.130 | > .20 | 26 | 0.128 | > .20 |
| Pb_L | 27 | 0.230 | < .10 | 19 | 0.224 | > .20 |
| Zn_L | 31 | 0.099 | > .20 | 26 | 0.092 | > .20 |

*^K^* ^Kidney,^ *^L^* ^Liver^

Table S4 - Normality of variances for metals in kidney and liver samples of golden jackals (Kolmogorov-Smirnov Tests)

| Element  and tissue | Adult male | | | Adult female | | | Juvenile male | | | Juvenile female | | |
| --- | --- | --- | --- | --- | --- | --- | --- | --- | --- | --- | --- | --- |
|  | N | d-value | p-value | N | d-value | p-value | N | d-value | p-value | N | d-value | p-value |

| Al_K | 21 | 0.115 | > .20 | 20 | 0.131 | > .20 | 0 |  |  | 0 |  |  |
| --- | --- | --- | --- | --- | --- | --- | --- | --- | --- | --- | --- | --- |
| Ca_K | 22 | 0.179 | > .20 | 22 | 0.121 | > .20 | 0 |  |  | 0 |  |  |
| Cd_K | 24 | 0.196 | > .20 | 21 | 0.223 | < .15 | 0 |  |  | 0 |  |  |
| Cr_K | 24 | 0.131 | > .20 | 22 | 0.119 | > .20 | 0 |  |  | 0 |  |  |
| Cu_K | 24 | 0.111 | > .20 | 22 | 0.210 | > .20 | 0 |  |  | 0 |  |  |
| Fe_K | 24 | 0.199 | > .20 | 22 | 0.126 | > .20 | 0 |  |  | 0 |  |  |
| K_K | 24 | 0.156 | > .20 | 22 | 0.102 | > .20 | 0 |  |  | 0 |  |  |
| Mg_K | 24 | 0.108 | > .20 | 22 | 0.130 | > .20 | 0 |  |  | 0 |  |  |
| Mn_K | 24 | 0.198 | > .20 | 21 | 0.108 | > .20 | 0 |  |  | 0 |  |  |
| Na_K | 24 | 0.091 | > .20 | 22 | 0.122 | > .20 | 0 |  |  | 0 |  |  |
| Pb_K | 20 | 0.179 | > .20 | 20 | 0.276 | < .10 | 0 |  |  | 0 |  |  |
| Zn_K | 24 | 0.103 | > .20 | 22 | 0.160 | > .20 | 0 |  |  | 0 |  |  |
| Al_L | 73 | 0.102 | > .20 | 47 | 0.132 | > .20 | 5 | 0.185 | > .20 | 12 | 0.197 | > .20 |
| Ca_L | 55 | 0.149 | < .20 | 38 | 0.212 | < .10 | 4 | 0.249 | > .20 | 13 | 0.176 | > .20 |
| Cd_L | 72 | 0.141 | < .15 | 44 | 0.159 | > .20 | 4 | 0.272 | > .20 | 5 | 0.206 | > .20 |
| Cr_L | 76 | 0.103 | > .20 | 46 | 0.062 | > .20 | 8 | 0.140 | > .20 | 13 | 0.210 | > .20 |
| Cu_L | 82 | 0.141 | < .10 | 49 | 0.110 | > .20 | 8 | 0.195 | > .20 | 14 | 0.210 | > .20 |
| Fe_L | 82 | 0.093 | > .20 | 50 | 0.130 | > .20 | 8 | 0.230 | > .20 | 14 | 0.142 | > .20 |
| K_L | 82 | 0.077 | > .20 | 50 | 0.057 | > .20 | 8 | 0.213 | > .20 | 14 | 0.220 | > .20 |
| Mg_L | 82 | 0.077 | > .20 | 50 | 0.105 | > .20 | 8 | 0.123 | > .20 | 14 | 0.149 | > .20 |
| Mn_L | 80 | 0.152 | < .10 | 50 | 0.089 | > .20 | 8 | 0.143 | > .20 | 13 | 0.126 | > .20 |
| Na_L | 80 | 0.122 | < .20 | 50 | 0.132 | > .20 | 8 | 0.276 | > .20 | 14 | 0.246 | > .20 |
| Pb_L | 64 | 0.208 | **< .01** | 42 | 0.230 | **< .05** | 7 | 0.292 | > .20 | 9 | 0.194 | > .20 |
| Zn_L | 82 | 0.122 | < .20 | 50 | 0.083 | > .20 | 8 | 0.206 | > .20 | 14 | 0.240 | > .20 |

*^K^* ^Kidney,^ *^L^* ^Liver; Bold marked tests are significant at p <.05^

Table S5 - Homogeneity of variances and between-species comparison of concentration values (mg/kg dw) for metals in kidney and liver samples of red foxes (Vulpes vulpes) and golden jackals (Canis aureus)

| Variable | Mean concentrations | | Homogeneity of variances | | | Between-species comparisons |
| --- | --- | --- | --- | --- | --- | --- |
|  | Fox | Jackal | Levene (W) | df | p | p |
| Al_K | 15.720 | 13.821 | 8.086 | 63 | **0.0060** | 0.5913* |
| Ca_K | 298.553 | 285.364 | 1.083 | 69 | 0.3017 | 0.7344** |
| Cd_K | 0.928 | 0.442 | 9.007 | 68 | **0.0038** | **0.0018*** |
| Cr_K | 0.388 | 0.322 | 0.366 | 68 | 0.5472 | 0.1653** |
| Cu_K | 11.870 | 14.625 | 2.642 | 71 | 0.1085 | **0.0268**** |
| Fe_K | 194.828 | 257.579 | 2.964 | 70 | 0.0896 | **0.0137**** |
| K_K | 8499.768 | 7941.743 | 1.180 | 71 | 0.2810 | 0.2509** |
| Mg_K | 540.443 | 550.145 | 0.871 | 70 | 0.3540 | 0.7474** |
| Mn_K | 4.579 | 5.195 | 1.178 | 70 | 0.2816 | 0.0768** |
| Na_K | 5525.143 | 5425.288 | 0.001 | 71 | 0.9828 | 0.8052** |
| Pb_K | 3.736 | 3.441 | 0.487 | 64 | 0.4877 | 0.6995** |
| Zn_K | 65.958 | 74.082 | 2.881 | 70 | 0.0941 | **0.0283**** |
| Al_L | 20.543 | 19.271 | 2.600 | 188 | 0.1085 | 0.4022** |
| Ca_L | 247.392 | 201.998 | 0.660 | 159 | 0.4178 | 0.1043** |
| Cd_L | 0.333 | 0.193 | 11.057 | 176 | **0.0011** | **0.0003*** |
| Cr_L | 0.511 | 0.452 | 14.322 | 195 | **0.0002** | 0.6590* |
| Cu_L | 40.860 | 58.744 | 6.558 | 207 | **0.0112** | **0.0011*** |
| Fe_L | 585.501 | 718.599 | 1.552 | 210 | 0.2143 | **0.0080**** |
| K_L | 7898.498 | 8123.838 | 1.847 | 210 | 0.1756 | 0.3618** |
| Mg_L | 503.627 | 488.793 | 1.484 | 209 | 0.2245 | 0.5649** |
| Mn_L | 10.415 | 11.352 | 1.698 | 207 | 0.1940 | 0.0719** |
| Na_L | 3872.336 | 3392.137 | 1.422 | 208 | 0.2344 | **0.0072**** |
| Pb_L | 7.414 | 5.531 | 6.494 | 166 | **0.0117** | 0.2469* |
| Zn_L | 112.229 | 110.646 | 2.231 | 209 | 0.1368 | 0.7285** |

^K Kidney, L Liver, df degree of freedom, p value of significance, * Mann-Whitney U Test, ** T-test for independent samples; Bold marked tests are significant at p <.05^

Table S6 – Liver vs. kidney comparisons between levels of selected element concentrations (mg/kg dw) in golden jackal samples, based on a higher number of data and kidney-liver pairs (organs of the same specimens)

| Vari-able | Full data set | | | | | | Data pairs | | | | |
| --- | --- | --- | --- | --- | --- | --- | --- | --- | --- | --- | --- |
|  | Liver | | Kidney | | Levene | L vs. K | N | Liver mean | Kidney mean | Levene | L vs. K |
|  | N | Mean | N | Mean | p | p |  |  |  | p | p |
| Al | 137 | 19.27 | 41 | 13.82 | **<0.001** | **<0.001^2^** | 30 | 15.05 | 13.83 | 0.082 | 0.268^3^ |
| Ca | 109 | 197.46 | 44 | 285.36 | 0.995 | **0.001^1^** | 27 | 217.39 | 280.98 | 0.801 | 0.083^3^ |
| Cd | 125 | 0.19 | 45 | 0.44 | **<0.001** | **<0.001^2^** | 30 | 0.19 | 0.46 | **0.001** | **<0.001^4^** |
| Cr | 143 | 0.45 | 46 | 0.32 | 0.494 | **<0.001^1^** | 35 | 0.36 | 0.30 | 0.241 | 0.082^3^ |
| Cu | 153 | 58.74 | 46 | 14.62 | **<0.001** | **<0.001^2^** | 37 | 38.78 | 14.70 | **<0.001** | **<0.001^4^** |
| Fe | 154 | 718.60 | 46 | 257.58 | **<0.001** | **<0.001^2^** | 37 | 721.73 | 261.02 | **<0.001** | **<0.001^4^** |
| K | 154 | 8123.84 | 46 | 7941.74 | 0.916 | 0.523^1^ | 37 | 8212.38 | 7956.31 | 0.378 | 0.453^3^ |
| Mg | 153 | 492.30 | 46 | 550.14 | **0.016** | **0.015^2^** | 37 | 598.67 | 545.65 | 0.250 | 0.063^3^ |
| Mn | 151 | 11.35 | 45 | 5.20 | **0.001** | **<0.001^2^** | 36 | 11.39 | 5.11 | **0.006** | **<0.001^4^** |
| Na | 152 | 3392.14 | 46 | 5425.29 | **0.002** | **<0.001^2^** | 36 | 3331.41 | 5412.91 | **0.001** | **<0.001^4^** |
| Pb | 122 | 5.53 | 41 | 3.77 | **0.008** | **0.019^2^** | 25 | 5.83 | 3.09 | **0.001** | 0.174^4^ |
| Zn | 154 | 110.65 | 46 | 74.08 | **0.006** | **<0.001^2^** | 37 | 104.11 | 73.81 | 0.643 | **<0.001^3^** |

^L – Liver,^ ^K – Kidney, 1 – T-test for Independent Samples; 2 – T-test for Independent samples with separate variance estimates; 3 – T-test for Dependent Samples; 4 – Wilcoxon Matched Pairs Test; Bold marked tests are significant at p <.05^

Table S7 – Liver vs. kidney comparisons between levels of selected element concentrations (mg/kg dw) in red fox samples based on a higher number of data and kidney-liver pairs (organs of the same specimens)

| Vari-able | Full data set | | | | | | Data pairs | | | | |
| --- | --- | --- | --- | --- | --- | --- | --- | --- | --- | --- | --- |
|  | Liver | | Kidney | | Levene | L vs. K | N | Liver mean | Kidney mean | Levene | L vs. K |
|  | N | Mean | N | Mean | p | p |  |  |  | p | p |
| Al | 53 | 20.54 | 24 | 15.72 | 0.066 | 0.054^1^ | 16 | 14.04 | 14.96 | 0.816 | 0.581^3^ |
| Ca | 51 | 247.39 | 27 | 298.55 | 0.853 | 0.236^1^ | 20 | 255.79 | 260.89 | 0.678 | 0.908^3^ |
| Cd | 53 | 0.33 | 25 | 0.93 | **<0.001** | **<0.001^2^** | 17 | 0.30 | 1.02 | **0.005** | **<0.001^4^** |
| Cr | 54 | 0.51 | 24 | 0.39 | 0.055 | 0.107^1^ | 17 | 0.38 | 0.40 | 0.446 | 0.787^3^ |
| Cu | 56 | 40.86 | 27 | 11.87 | **<0.001** | **<0.001^2^** | 21 | 39.23 | 12.36 | **<0.001** | **<0.001^4^** |
| Fe | 58 | 585.50 | 26 | 194.83 | **<0.001** | **<0.001^2^** | 20 | 711.45 | 201.95 | **<0.001** | **<0.001^4^** |
| K | 58 | 7898.50 | 27 | 8499.77 | **0.044** | 0.227^2^ | 21 | 7740.36 | 8646.05 | 0.160 | 0.120^3^ |
| Mg | 58 | 503.63 | 26 | 540.44 | **0.035** | 0.209^2^ | 20 | 579.08 | 540.40 | 0.681 | 0.232^3^ |
| Mn | 58 | 10.42 | 27 | 4.58 | **<0.001** | **<0.001^2^** | 21 | 10.35 | 4.59 | **0.015** | **<0.001^4^** |
| Na | 58 | 3872.34 | 27 | 5525.14 | 0.185 | **<0.001^1^** | 21 | 3649.62 | 5677.60 | **0.022** | **<0.001^4^** |
| Pb | 46 | 7.41 | 26 | 3.74 | **0.002** | **0.008^2^** | 17 | 10.95 | 3.58 | **<0.001** | **0.011^4^** |
| Zn | 57 | 112.23 | 26 | 65.96 | **<0.001** | **<0.001^2^** | 20 | 113.60 | 67.28 | **0.005** | **<0.001^4^** |

^L – Liver, K – Kidney, 1 – T-test for Independent Samples; 2 –^ ^T-test for Independent samples with separate variance estimates; 3 – T-test for Dependent Samples; 4 – Wilcoxon Matched Pairs Test; Bold marked tests are significant at p <.05^

Table S8 - Effects of sex factor on concentrations of selected elements (mg/kg dw) in kidney tissues of golden jackals

| Element | Female | | Male | | T-test | | | Levene-test | |
| --- | --- | --- | --- | --- | --- | --- | --- | --- | --- |
|  | Valid N | Mean | Valid N | Mean | t-value | df | p | W | p |
| Al_K | 20 | 12.619 | 21 | 14.966 | -2.047 | 39 | **0.047** | 2.027 | 0.162 |
| Ca_K | 22 | 247.283 | 22 | 323.445 | -1.821 | 42 | 0.076 | 8.639 | 0.005 |
| Cd_K | 21 | 0.427 | 24 | 0.455 | -0.251 | 43 | 0.803 | 0.454 | 0.504 |
| Cr_K | 22 | 0.302 | 24 | 0.340 | -0.725 | 44 | 0.473 | 0.613 | 0.438 |
| Cu_K | 22 | 14.852 | 24 | 14.416 | 0.263 | 44 | 0.794 | 2.578 | 0.116 |
| Fe_K | 22 | 231.355 | 24 | 281.617 | -1.578 | 44 | 0.122 | 8.716 | 0.005 |
| K_K | 22 | 7743.266 | 24 | 8123.681 | -0.739 | 44 | 0.464 | 3.525 | 0.067 |
| Mg_K | 22 | 528.039 | 24 | 570.408 | -1.115 | 44 | 0.271 | 0.415 | 0.523 |
| Mn_K | 21 | 4.733 | 24 | 5.600 | -2.020 | 43 | **0.050** | 0.074 | 0.787 |
| Na_K | 22 | 5364.275 | 24 | 5481.216 | -0.242 | 44 | 0.810 | 1.385 | 0.246 |
| Pb_K | 20 | 3.177 | 21 | 4.341 | -1.058 | 39 | 0.296 | 2.024 | 0.163 |
| Zn_K | 22 | 68.108 | 24 | 79.559 | -2.529 | 44 | **0.015** | 0.014 | 0.906 |

^Bold marked tests are significant at p <.05 (t-test for independent samples, by groups)^

Table S9 - Effects of sex factor on concentrations of selected elements (mg/kg dw) in kidney and liver tissues of red foxes

| Element | Female | | Male | | T-test | | | Levene-test | |
| --- | --- | --- | --- | --- | --- | --- | --- | --- | --- |
|  | Valid N | Mean | Valid N | Mean | t-value | df | p | W | p |
| Al_K | 12 | 15.820 | 12 | 15.620 | 0.068 | 22 | 0.946 | 0.000 | 0.991 |
| Ca_K | 14 | 295.012 | 13 | 302.368 | -0.103 | 25 | 0.919 | 0.304 | 0.586 |
| Cd_K | 13 | 1.183 | 12 | 0.651 | 1.920 | 23 | 0.067 | 3.101 | 0.092 |
| Cr_K | 12 | 0.458 | 12 | 0.317 | 1.768 | 22 | 0.091 | 0.303 | 0.588 |
| Cu_K | 14 | 12.752 | 13 | 10.919 | 1.218 | 25 | 0.235 | 0.039 | 0.845 |
| Fe_K | 13 | 189.091 | 13 | 200.566 | -0.345 | 24 | 0.733 | 3.287 | 0.082 |
| K_K | 14 | 8975.913 | 13 | 7986.997 | 1.091 | 25 | 0.286 | 1.028 | 0.320 |
| Mg_K | 13 | 554.524 | 13 | 526.362 | 0.652 | 24 | 0.521 | 0.586 | 0.452 |
| Mn_K | 14 | 4.796 | 13 | 4.345 | 0.920 | 25 | 0.367 | 0.754 | 0.393 |
| Na_K | 14 | 5987.475 | 13 | 5027.247 | 1.467 | 25 | 0.155 | 3.211 | 0.085 |
| Pb_K | 14 | 3.846 | 12 | 3.607 | 0.182 | 24 | 0.857 | 1.219 | 0.280 |
| Zn_K | 13 | 69.276 | 13 | 62.641 | 1.474 | 24 | 0.154 | 0.162 | 0.691 |
| Al_L | 24 | 19.275 | 29 | 21.591 | -0.756 | 51 | 0.453 | 0.091 | 0.764 |
| Ca_L | 22 | 275.394 | 29 | 226.148 | 0.973 | 49 | 0.335 | 0.407 | 0.526 |
| Cd_L | 23 | 0.413 | 30 | 0.271 | 2.006 | 51 | 0.050 | 0.135 | 0.715 |
| Cr_L | 25 | 0.496 | 29 | 0.524 | -0.291 | 52 | 0.772 | 0.184 | 0.670 |
| Cu_L | 25 | 47.103 | 31 | 35.826 | 1.485 | 54 | 0.143 | 1.827 | 0.182 |
| Fe_L | 26 | 599.288 | 32 | 574.299 | 0.337 | 56 | 0.737 | 0.180 | 0.673 |
| K_L | 26 | 8027.642 | 32 | 7793.568 | 0.645 | 56 | 0.522 | 0.523 | 0.472 |
| Mg_L | 26 | 519.127 | 32 | 491.033 | 0.707 | 56 | 0.483 | 1.772 | 0.189 |
| Mn_L | 26 | 10.803 | 32 | 10.100 | 0.726 | 56 | 0.471 | 0.699 | 0.407 |
| Na_L | 26 | 3734.150 | 32 | 3984.612 | -0.758 | 56 | 0.452 | 0.855 | 0.359 |
| Pb_L | 19 | 9.017 | 27 | 6.286 | 1.140 | 44 | 0.260 | 1.221 | 0.275 |
| Zn_L | 26 | 118.059 | 31 | 107.340 | 1.292 | 55 | 0.202 | 0.652 | 0.423 |

*^K^* ^Kidney,^ *^L^* ^Liver^

Table S10 – Effects of sex, age group and sex*age group factors on concentrations of selected elements in liver tissues of golden jackals

| Element | Concentrations of selected elements (mg/kg dw) | | | | | | df | p-values of ANOVA tests | | |
| --- | --- | --- | --- | --- | --- | --- | --- | --- | --- | --- |
|  | Adult | | | Juvenile | | |  |  |  |  |
|  | Female | Male | Total | Female | Male | Total |  | Sex | Age group | Sex*Age group |
| Al | 17.54 | 20.25 | 19.19 | 19.77 | 20.11 | 19.87 | 133 | 0.532 | 0.667 | 0.627 |
| Ca | 163.38 | 199.15 | 184.38 | 304.24 | 151.33 | 268.26 | 105 | 0.190 | 0.298 | **0.036** |
| Cd | 0.23 | 0.18 | 0.20 | 0.11 | 0.15 | 0.13 | 121 | 0.975 | 0.202 | 0.464 |
| Cr | 0.41 | 0.46 | 0.44 | 0.50 | 0.48 | 0.49 | 139 | 0.787 | 0.293 | 0.450 |
| Cu | 61.03 | 49.26 | 53.66 | 105.54 | 60.11 | 89.02 | 149 | **0.001** | **0.002** | 0.054 |
| Fe | 682.37 | 748.49 | 723.44 | 673.74 | 717.17 | 689.53 | 150 | 0.501 | 0.806 | 0.889 |
| K | 7897.77 | 8005.53 | 7964.71 | 9446.84 | 8434.20 | 9078.60 | 150 | 0.250 | **0.013** | 0.155 |
| Mg | 515.74 | 472.69 | 489.12 | 543.85 | 454.04 | 511.20 | 149 | 0.093 | 0.848 | 0.624 |
| Mn | 12.06 | 10.64 | 11.19 | 12.49 | 12.19 | 12.38 | 147 | 0.264 | 0.199 | 0.462 |
| Na | 3344.89 | 3415.04 | 3388.06 | 3593.08 | 3106.70 | 3416.21 | 148 | 0.436 | 0.910 | 0.298 |
| Pb | 5.39 | 5.37 | 5.38 | 8.20 | 4.40 | 6.54 | 118 | 0.199 | 0.536 | 0.205 |
| Zn | 114.47 | 104.86 | 108.50 | 131.29 | 109.98 | 123.54 | 150 | **0.021** | 0.101 | 0.380 |

^Bold marked tests are significant at p <.05^

Table S11 – Heavy metal (H.m) concentrations in comparison with toxic and nontoxic levels in liver and kidney samples of canine species (mg/kg dw)

|  | Present study | | | |  | Literature data for threshold values | | | | | | |
| --- | --- | --- | --- | --- | --- | --- | --- | --- | --- | --- | --- | --- |
| H.m. | Liver | | Kidney | | Source | Species or family group | Liver | | | Kidney | | |
|  | Jackal | Fox | Jackal | Fox |  |  | Normal | High | Toxic | Normal | High | Toxic |
| Cd | 0.19 | 0.33 | 0.44 | 0.93 | (Tomza-Marciniak et al. 2019) | mammals | n.a. | n.a. | n.a. | <350 | n.a. | n.a. |
| Cu | 58.7 | 40.9 | 14.6 | 11.9 | (Puls 1994) | domestic dog | 90-300^*^ | n.a. | 1200-9000^*^ | 12.5-37.5^*^ | n.a. | >50^*^ |
| Fe | 718.6 | 585.5 | 257.6 | 194.8 | (Kosik-Bogacka et al. 2019) | canines | 330-1000 | n.a. | >1700 | 330-750 | n.a. | n.a. |
| Mn | 11.4 | 10.4 | 5.2 | 4.6 | (Kalisińska and Budis 2019) | canines | 10-16.7 | n.a. | n.a. | 6.0-9.0 | n.a. | n.a. |
| Pb | 5.5 | 7.4 | 3.4 | 3.7 | (Baranowska-Bosiacka et al. 2019) | canines | 0.9-35.1^*^ | 36-51^*^ | 501-2001^*^ | 1.0-25.0^*^ | 50-100^*^ | 100-500^*^ |
| Zn | 110.6 | 112.2 | 74.1 | 66.0 | (Kosik-Bogacka and Łanocha-Arendarczyk 2019) | canines | 100-233 | n.a. | >1233 | 80-150 | n.a. | >1500 |

^n.a. - * - Converted threshold values from wet weight to dry weight using conversion factors (CF = 3.0 for liver; CF = 2.5 for kidney) proposed by^ ^Kalisińska (2019a)^
